# Isolation of Potato Endophytes and Screening of *Chaetomium globosum* Antimicrobial Genes

**DOI:** 10.3390/ijms23094611

**Published:** 2022-04-21

**Authors:** Jiaxin Zhang, Md. Samiul Islam, Jieyu Wang, Yang Zhao, Wubei Dong

**Affiliations:** Department of Plant Pathology, College of Plant Science and Technology and the Key Lab of Crop Disease Monitoring & Safety Control in Hubei Province, Huazhong Agricultural University, Wuhan 430070, China; 2019301110162@webmail.hzau.edu.cn (J.Z.); samiulislam@webmail.hzau.edu.cn (M.S.I.); 2019301120134@webmail.hzau.edu.cn (J.W.); yzhao@webmail.hzau.edu.cn (Y.Z.)

**Keywords:** endophyte, potato, *Chaetomium globosum*, antimicrobial peptide, *Bacillus subtilis*

## Abstract

Antimicrobial peptides (AMPs) have natural antibacterial activities that pathogens find difficult to overcome. As a result of this occurrence, AMPs can act as an important substitute against the microbial resistance. In this study, we used plate confrontation tests to screen out 20 potential endophytes from potato tubers. Among them, endophyte F5 was found to significantly inhibit the growth of five different pathogenic fungi. Following that, phylogenetic analysis revealed that the internal transcribed spacer (ITS) sequences were 99% identical to *Chaetomium globosum* corresponding sequences. Thereafter, the *Bacillus subtilis* expression system was used to create a *C. globosum* cDNA library in order to isolate the resistance genes. Using this approach, the resistance gene screening technology in the indicator bacteria built-in library was used to identify two antimicrobial peptides, CgR2150 and CgR3101, with broad-spectrum antibacterial activities. Furthermore, the results showed that CgR2150 and CgR3101 have excellent UV, thermal, and enzyme stabilities. Also, these two peptides can significantly inhibit the growth of various bacteria (*Xanthomonas oryzae* pv. oryzae, *Xanthomonas oryzae* pv. oryzicola, *Clavibacter michiganensis*, and *Clavibacter fangii*) and fungi (*Fusarium graminearum*, *Rhizoctonia solani*, and *Botrytis cinerea*). Scanning electron microscopy (SEM) observations revealed that CgR2150 and CgR3101 peptides act against bacteria by disrupting bacterial cell membranes. Moreover, hemolytic activity assay showed that neither of the two peptides exhibited significant hemolytic activity. To conclude, the antimicrobial peptides CgR2150 and CgR3101 are promising in the development of a new antibacterial agent and for application in plant production.

## 1. Introduction

Microorganisms, including bacteria, fungi, and viruses, have always posed a threat to human, animal, and plant health. Antibiotics have effectively treated a wide range of diseases to lessen the damage. However, the overuse of antibiotics has resulted in the development of drug resistance in many pathogenic microbes, which will have disastrous consequences for humans and the biosphere [1,2]. To solve this problem, researchers have been looking for novel antibacterial compounds. Currently, antimicrobial peptides (AMPs) are a powerful tool against the microbial resistant pattern because they have several advantages, such as high activity, low drug resistance, and multiple modes of action, and they can resist infection by external pathogenic microorganisms by affecting the host’s immune response or directly inhibiting or killing some pathogenic bacteria, fungi, parasites, and viruses [3,4].

AMPs are naturally occurring active molecules produced by the organism. They are normally made up of 12 to 100 amino acids and have a helix or sheet structure. Besides, their structures are generally cyclic, hydrophobic, and include unique moieties such as D-amino acids (AA), which is an important prerequisite for the use as a bioactive candidate [5]. AMPs can be found in a wide range of organisms, including bacteria, fungi, higher plants, insects and other invertebrates, amphibians, fish, mammals, and even humans [6,7,8,9]. Furthermore, these peptides have antibacterial, antifungal, antiviral, and anti-parasitic features, as well as anti-inflammatory and anti-tumor properties, which makes them useful in agriculture, animal husbandry, aquaculture, and medicine [10,11,12,13,14]. In addition, AMPs have a variety of mechanisms of action. The most common action involves membrane ion channel formation and disruption via electrostatic attraction, resulting in intracellular substance leakage and cell death. Hence, these phenomena make it difficult for pathogens to develop drug resistance [15,16].

The prokaryotic expression system is one of the most frequently utilized genetic engineering approaches for the expression and secretion of exogenous target genes [17]. *Bacillus subtilis* has been one of the most extensively utilized prokaryotic expression systems in recent years due to its rapid growth, absence of endotoxin, and minimal damage to organism [18]. Moreover, *B. subtilis* possesses a robust secretion and expression capability, which enables it to directly secrete recombinant proteins into the extracellular space, thereby contributing significantly to the large-scale development of antibacterial genes [19,20,21]. Using *B. subtilis* as a bioengineering strain to generate functional protein products has the potential to reduce costs and add economic values [22,23].

Potato is the fourth largest food crop in the world after corn, rice, and wheat. It is primarily propagated via tubers, thereby the endophytes can be transmitted directly. In recent years, secondary metabolites such as alkaloids, lignins, terpenoids, and anthraquinones have been isolated from a variety of endophytic fungi, with the majority of them having antibacterial activity [24,25,26]. *Chaetomium globosum* is a widespread endophytic fungus found in Buxus L, Holly, Ginkgo, and Longan trees according to the reports [27]. *C. globosum* is a type of biological control agent with application potential that can prevent and treat a variety of plant diseases [28,29]. It can also produce a wide range of secondary metabolites with biological activity, including chaetocin, ergosterol, erpenes, cellulase, and so on. These secondary metabolites not only have good inhibitory activity against a range of microbes such as *Rhizoctonia solani*, *Pyricularia oryzae*, *Anthracnose*, and *Phytophthora parasitica*, but also have anti-tumor, anti-malarial, and anti-inflammatory properties [30,31,32].

In our laboratory, we developed a method for screening potential antimicrobial genes using a *B. subtilis* expression system. Using this technology, resistance genes have been successfully isolated from rice, pinellia, maize, and potatoes [23,33,34,35]. In this study, *C. globosum* was isolated and screened from potato tubers as an endophytic fungus with a significant antagonistic effect on pathogenic fungi. The *B. subtilis* expression system was used to screen out genes of antimicrobial peptides from *C. globosum* with broad-spectrum antibacterial activities. Then, the physicochemical properties and bacteriostatic capability of the obtained peptides were investigated, establishing the foundation for future research into the function and application of *C. globosum* resistance genes.

## 2. Results

### 2.1. Isolation, Purification, and Screening of Potato Endophytes

**Figure 1:**
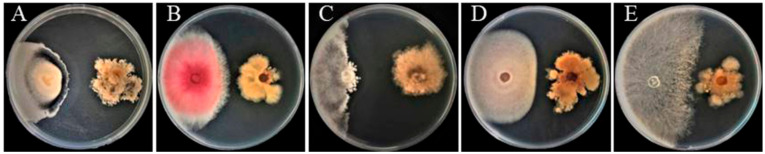
Endophyte F5 inhibits the growth of five pathogenic fungi’s mycelia. Inhibitory effect on (**A**) *V. dahlia,* (**B**) *F. graminearum*, (**C**) *B. cinerea*, (**D**) *F. oxysporum*, (**E**) *R. solani*.

A total of 20 endophytic fungi were isolated from potato tubers and named as F1-F20. Through the plate confrontation experiment, the endophytic fungus antagonistic effect was screened against *Fusarium oxysporum*, *Rhizoctonia solani*, *Fusarium graminearum*, *Verticillium dahliae,* and *Botrytis cinerea*. *B. cinerea* had the highest inhibition rate of 78.82 percent, followed by other fungi (the inhibition rate against *R. solani* is 61.91%; *F. graminearum*, 54.48%; and *V. dahliae*, 48.68%) (Figure 1). These findings suggest that the endophyte F5 can suppress the growth of several pathogenic fungi, suggesting that further research is necessary.

### 2.2. Identification of Endophytic Fungus F5

**Figure 2:**
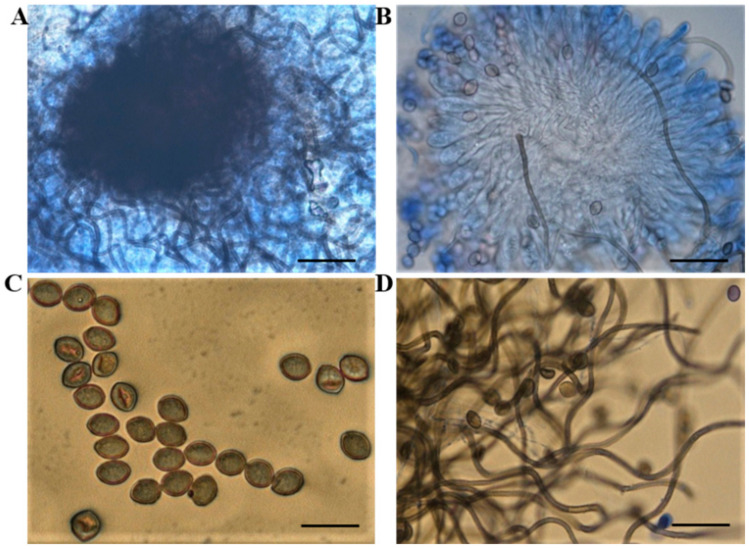
Microscopic observation of endophyte F5 under light microscope. (**A**) perithecium; (**B**) ascus; (**C**) ascospores, and (**D**) appendage. Bars: A = 80 μm, B = 60 μm, C = 20 μm, and D = 40 μm.

**Figure 3:**
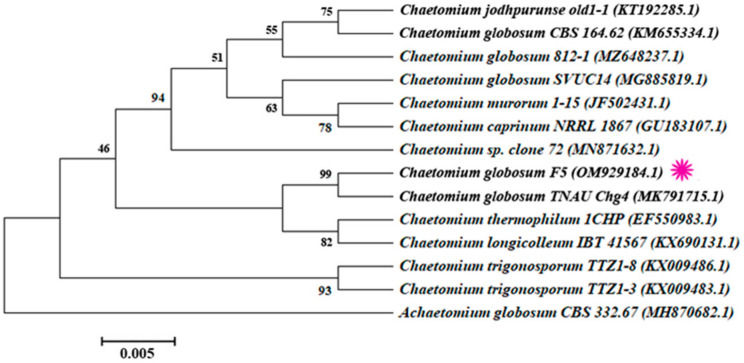
Phylogenetic tree of *Chaetomium globosum* based on 5.8S rRNA gene sequencing showing similarity with *Chaetomium* spp. The Neighbor-Joining (NJ) approach was used in MEGA7, with bootstrap values (*n* = 1000) greater than 50% apparent at the tree’s internodes. As the outer group, *Achaetomium globosum* CBS 332.67 strain was included. The purple symbol indicates the position of our isolated endophyte strain (F5).

The morphological features of F5 were obtained with the following characteristics: yellowish-brown with a wave-shaped edge of the colony, with olive-brown or gray-white aerial hyphae, often with olive-colored exudates; the ascus shells are superficial, spherical or nearly spherical, with dense appendages in outer layers; the asci are clustered, stalked, cudgel-shaped, with eight ascospores inside; the ascospores are lemon-shaped or oval (Figure 2). In addition, the 5.8S rRNA sequence fragment of endophytic fungus F5 was amplified using the universal primers ITS1 and ITS4, and the target size was obtained about 500 bp (Figure S1). The F5 isolates’ sequences were submitted to the GenBank database, where they were assigned the accession number OM929184.1 (Table S1). Following an evolutionary analysis of the ITS sequences, the phylogenetic analysis revealed that this new isolate had the highest similarity to *Chaetomium globosum* TNAU Chg4 from the GenBank database (99%) (GenBank accession number MK791715) (Figure 3). As a result of the morphological and molecular analyses, we confirmed that our isolated endophyte F5 was *C. globosum*. 

### 2.3. Screening of Antibacterial Genes from C. globosum cDNA Library by B. subtilis System

**Figure 4:**
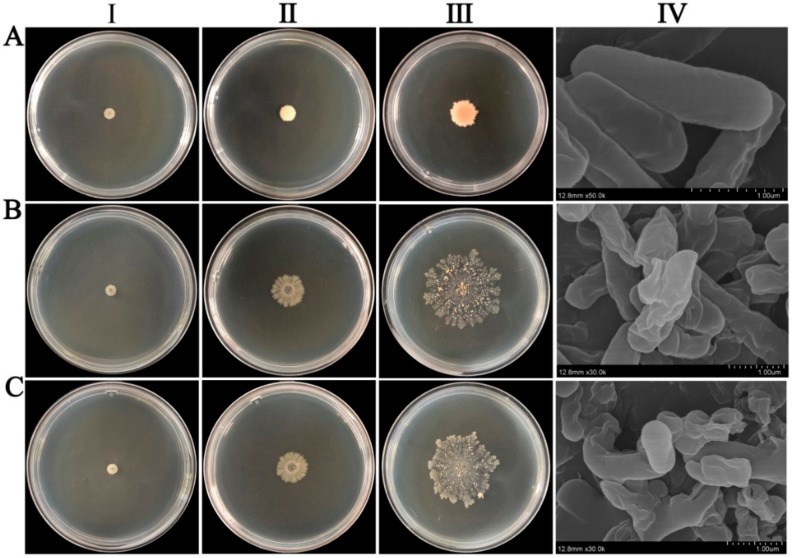
Injury effect of antimicrobial genes of *C. globosum* on *B. subtilis* cells. *B. subtilis* strains (SCK6-e) containing the empty vector and autolytic strains (CgR2150 and CgR3101) were placed on Luria-Bertani (LB) plates and incubated at 37 °C. (**A-I**) *B. subtilis* SCK6-e strain, (**B-I**) *CgR2150* and (**C-I**) *CgR3101* after 12 h of incubation, and (**A-II**) *B. subtilis* SCK6-e strain, (**B-II**) *CgR2150*, and (**C-II**) *CgR3101* after 48 h of incubation, while (**A-III**) *B. subtilis* SCK6-e strain, (**B-III**) *CgR2150*, and (**C-III**) *CgR3101* after 192 h of incubation. (**A-IV**) *B. subtilis* SCK6-e strain, (**B-IV**) *CgR2150*, and (**C-IV**) *CgR3101* were observed under a scanning electron microscope.

There were 4280 transformants in the *C. globosum* cDNA library constructed using the *B. subtilis* expression system. Through detection, it was found that the recombination rate of the library was 92.4%, indicating that the quality of the library was good (Figure S3). From 4280 transformants, we screened 10 monoclonal strains with abnormal phenotype, which showed obvious autolysis (Figure 4). The electron microscopy results indicated that the control *B. subtilis* SCK6-e strain had normal cell morphology. However, when *CgR2150* and *CgR3101* genes were introduced into cells, the cell membrane was damaged, resulting in obvious shrinkage, deformation, and lysis. These findings reveal that when the exogenous resistance gene of *C. globosum* is introduced into the host cell, the expressed and secreted product has a toxic effect on the host cell, causing severe damage and autolysis. By performing blastp alignment analysis, we discovered that CgR2150 and CgR3101 have no homologs in the NCBI database, indicating that these two peptides are novel. On this basis, *CgR2150* and *CgR3101* genes were selected for further research.

### 2.4. Antibacterial Activities of Antimicrobial Genes against Pathogenic Bacteria and Fungi

**Figure 5:**
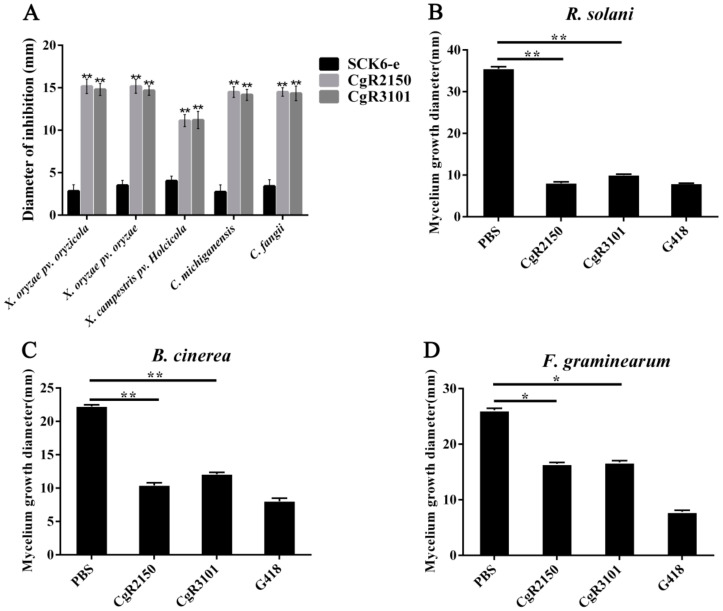
Determination of antimicrobial activities of two resistant genes against pathogenic bacteria and fungi. The *B. subtilis* SCK6-e strain was used as a control to determine the inhibitory effects of the peptides on pathogenic bacteria. Phosphate buffered saline (PBS) was used as a negative control, and geneticin (G418) was used as a positive control to detect the inhibitory activities of the peptides against pathogenic fungi. (**A**) Inhibitory effects of peptides CgR2150 and CgR3101 on five tested strains compared with SCK6-e. (**B**) Inhibitory activities of peptides CgR2150 and CgR3101 on *R. solani* hyphae compared to PBS buffer. (**C**) Inhibitory effects of peptides CgR2150 and CgR3101 on *B. cinerea* hyphae. (**D**) Inhibitory activities of peptides CgR2150 and CgR3101 on *F. graminearum* hyphae. The data are the mean values obtained after three independent experiments, and the vertical bars are the standard deviations (SD). Significance analysis was performed by *t*-test; * *p* < 0.05, ** *p* < 0.01.

Extracellular proteins from autolytic strains were extracted by ammonium sulfate precipitation method for antibacterial studies (Table S3). Compared with the control *B. subtilis* SCK6-e strain, the peptides CgR2150 and CgR3101 can significantly inhibit the growth of *Clavibacter fangii*, *Clavibacter michiganensis*, *Xanthomonas oryzae* pv. oryzae, *Xanthomonas oryzae* pv. oryzicola, and *Xanthomonas campestris* pv. Holcicola (Figure 5A). In addition, by measuring the inhibitory effects of peptides CgR2150 and CgR3101 on pathogenic fungi, it was found that CgR2150 and CgR3101 could significantly inhibit the mycelia growth of *R*. *solani*, *B*. *cinerea*, and *F*. *graminearum* (Figure 5B–D). The inhibitory activities of peptides CgR2150 and CgR3101 on tomato plant-infected diseases were determined under in vitro conditions at 7 d (Figure S4). The results showed that the incidence of tomato plants inoculated with *C. michiganensis* was significantly reduced after treatment with the two peptides (Table 1), indicating that the peptides CgR2150 and CgR3101 could effectively control the occurrence of *C. michiganensis*. From the above findings, it can be concluded that these two peptides had strong antibacterial and antifungal activities.

### 2.5. Effects of UV, Temperature, and Enzymes on Antibacterial Activities of Antimicrobial Peptides

**Figure 6:**
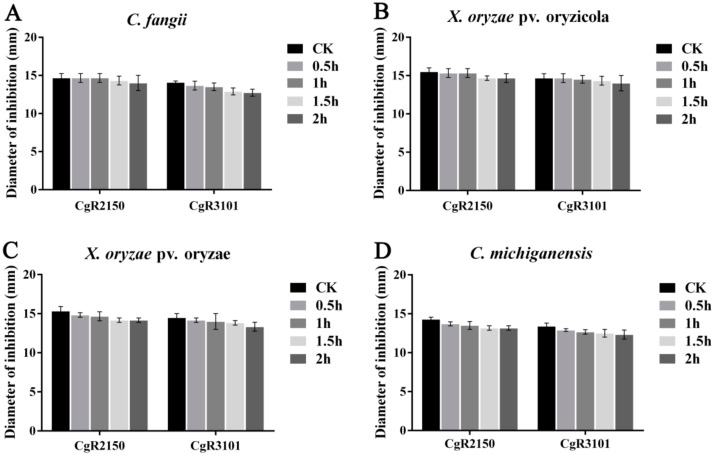
UV stability tests of CgR2150 and CgR3101. Antimicrobial peptides without UV treatment were used as controls. (**A**) *C. fangii*, (**B**) *X. oryzae* pv. oryzicola, (**C**) *X. oryzae* pv. oryzae, and (**D**) *C. michiganensis* are the antibacterial activity of the two peptides under UV treatment at different times (0.5 h, 1 h, 1.5 h, and 2 h). The data are the mean values obtained after three independent experiments, and the vertical bars are the standard deviations (SD). Significance analysis was performed by *t*-test.

**Figure 7:**
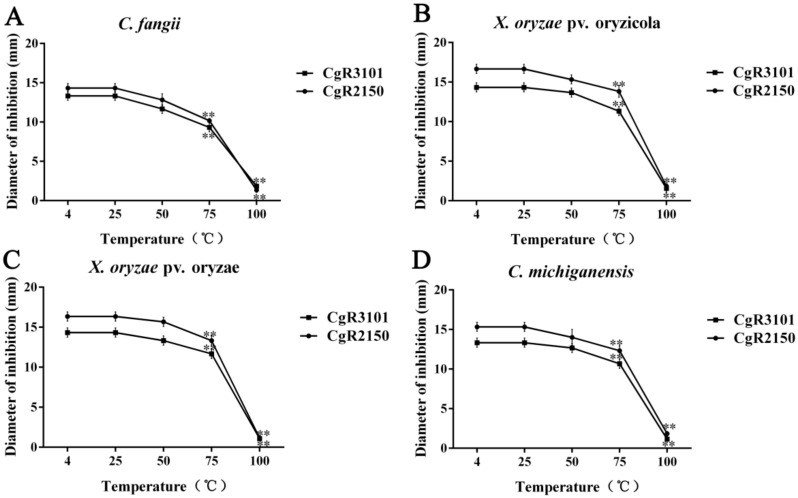
Thermal stability tests of CgR2150 and CgR3101. Antimicrobial peptides without temperature treatment at 4 °C were used as controls. (**A**) *C. fangii*, (**B**) *X. oryzae* pv. oryzicola, (**C**) *X. oryzae* pv. oryzae, and (**D**) *C. michiganensis* are the bacteriostatic diameter of the two peptides at different temperatures (25 °C, 50 °C, 75 °C, and 100 °C). The data are the mean values obtained after three independent experiments, and the vertical bars are the standard deviations (SD). Significance analysis was performed by *t*-test; ** *p* < 0.01.

**Figure 8:**
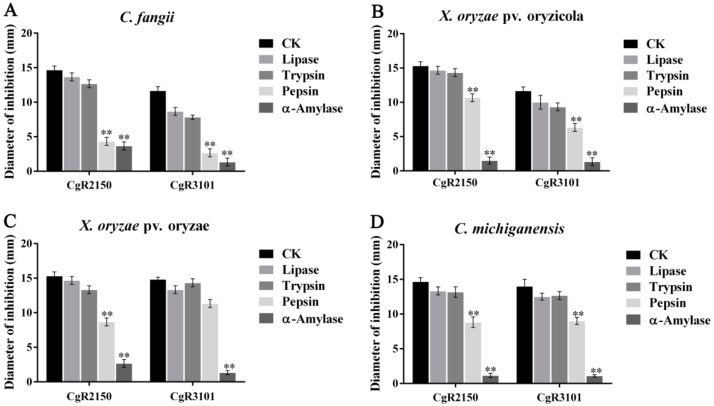
Enzyme stability assay of CgR2150 and CgR3101. Using the antimicrobial peptide without protease treatment as a control, the changes in the antimicrobial activity of the two peptides were observed through different biological enzyme treatments. (**A**) *C. fangii*, (**B**) *X. oryzae* pv. oryzicola, (**C**) *X. oryzae* pv. oryzae, and (**D**) *C. michiganensis* are the bacteriostatic diameter of two peptides treated with four different proteases, including lipase, trypsin, pepsin, and α-amylase. The data are the mean values obtained after three independent experiments, and the vertical bars are the standard deviations (SD). Significance analysis was performed by *t*-test; ** *p* < 0.01.

The two peptides were exposed to UV light for 0.5 h, 1 h, 1.5 h, and 2 h, respectively, to determine the stability of their antibacterial activity. The results showed that CgR2150 and CgR3101 exhibited stable bacteriostatic activity against Gram-positive and Gram-negative bacteria (Figure 6). For thermal stability testing, the antimicrobial peptides were heated at 25 °C, 50 °C, 75 °C, and 100 °C for 30 min. The results showed that CgR2150 and CgR3101 exhibited obvious antibacterial activity in the range of 4–75 °C; at 100 °C, both peptides lost their activity (Figure 7). In addition, by measuring the sensitivity of antimicrobial peptides to enzymes, it can be seen that CgR2150 and CgR3101 still have significant activities after treatment with trypsin and lipase (Figure 8). To conclude, the antimicrobial peptides CgR2150 and CgR3101 exhibit excellent UV, thermal, and enzymatic stability, laying the foundation for antimicrobial peptide application.

### 2.6. SDS-PAGE Analysis and Western Blot Reveal the Expression of Antimicrobial Peptides

**Figure 9:**
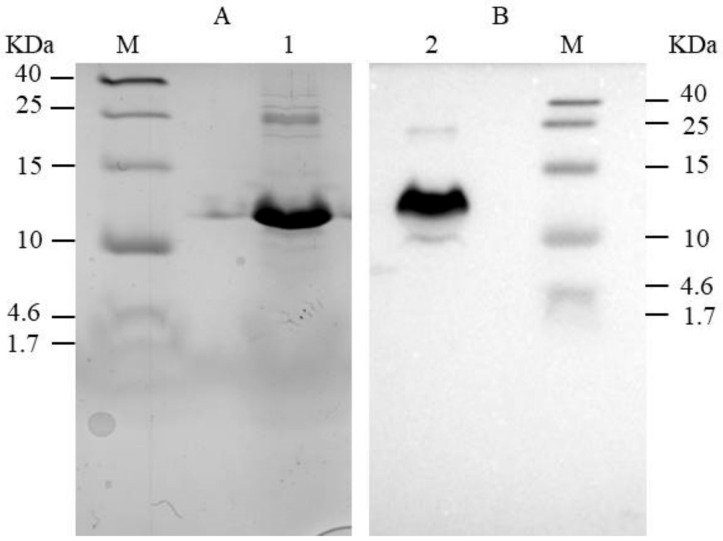
SDS-PAGE and western blotting analysis of fusion peptide CgR2150. (**A**) SDS-PAGE electrophoresis, (**B**) western blotting analysis. Lane M indicates pre-stained ultra-low molecular weight marker (1.7–40 kDa). Lanes 1 and 2 indicate the molecular weight of peptide CgR2150.

The His-tagged and TEV fusion CgR2150 and CgR3101 peptides were expressed in *B. subtilis* SCK6-e. To observe antimicrobial gene expression in host cells, we purified extracellular peptides using nickel columns and then detected their expression using SDS-PAGE and western blotting (Figure 9). The molecular weight of CgR2150 (approximately 12.5 kDa) can be observed from the SDS-PAGE gel and polyvinylidene fluoride (PVDF) membrane. The observed band is comparable in size to the predicted result from the website, indicating that the target protein was successfully expressed.

We firstly measured the amino acid sequences of CgR2150 and CgR3101 peptides, and then predicted their molecular weight, secondary structure, and isoelectric point (Table 2 and Table 3). The results showed that the CgR2150 peptide contained an α-helix structure. In addition, both CgR2150 and CgR3101 had higher isoelectric points, so we speculated that the antibacterial activity of the antimicrobial peptides might be related to the high electric point.

### 2.7. Minimum Inhibitory Concentration (MIC) Assay for Peptides CgR2150 and CgR3101

**Figure 10:**
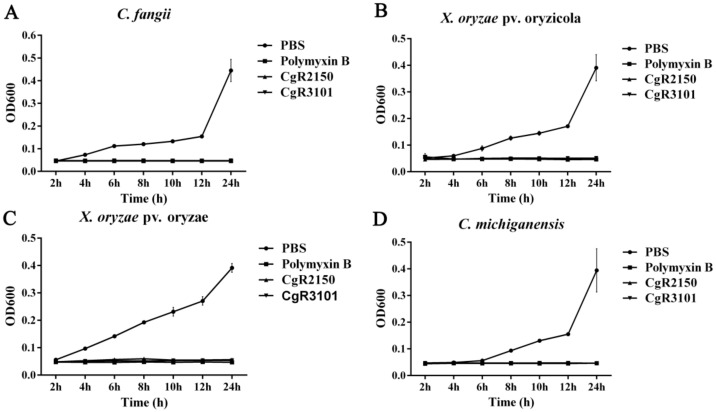
Time-kill curve of CgR2150 and CgR3101. The inhibitory effects of CgR2150 and CgR3101 on bacteria were demonstrated by bacterial growth curves. Among them, PBS buffer treatment was used as a negative control, and polymyxin B treatment was used as a positive control. (**A**) *C. fangii*, (**B**) *X. oryzae* pv. oryzicola, (**C**) *X. oryzae* pv. oryzae, and (**D**) *C. michiganensis* are time-kill kinetics by CgR2150 and CgR3101 at their MICs. The data are the mean values obtained after three independent experiments, and the vertical bars are the standard deviations (SD).

To observe the antibacterial activity of the peptides CgR2150 and CgR310, the minimum inhibitory concentration (MIC) values of the purified two peptides against Gram-negative bacteria and Gram-positive bacteria were determined. According to the findings, the peptides CgR2150 (10–25 µg/mL) and CgR3101 (10–24 µg/mL) had broad-spectrum antibacterial activity, considerably suppressed the pathogenic bacteria growth at lower concentrations, and had robust antibacterial activity against both Gram-negative and Gram-positive bacteria (Table 4). Besides, in order to further observe the killing effect of CgR2150 and CgR3101 on bacterial cells at MICs, we performed time-kill curve determination. The results showed that the two peptides had strong bactericidal effects on the four tested strains, and the bacteria hardly grew after 12 h of peptides treatment (Figure 10).

### 2.8. Hemolytic Activity Assay of CgR2150 and CgR3101 on Mammalian Cells

**Figure 11:**
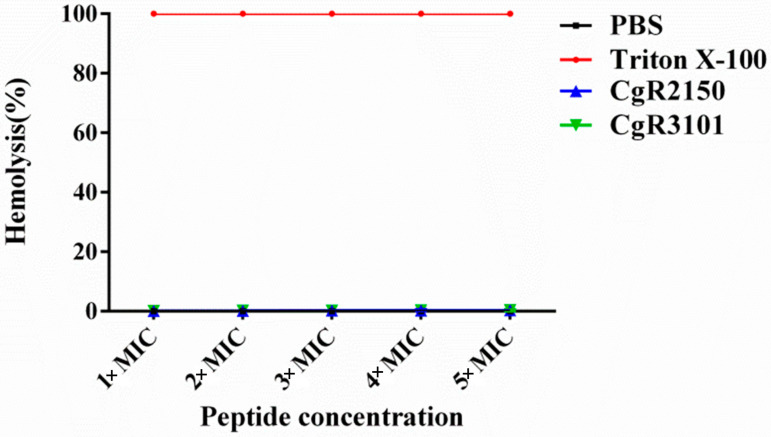
Hemolytic activities of the peptides on mammalian porcine erythrocytes.

To determine the hemolysis of antimicrobial peptides on mammalian cells, triton X-100 was used as a positive control (100%) and PBS buffer as a negative control (0%) (Figure 11). Then, the porcine erythrocytes were treated with CgR2150 and CgR3101, respectively. The results showed that CgR2150 and CgR3101 had minimal hemolytic activity in mammals, even at 5× MIC concentration, with hemolytic activities of 0.32% and 0.46%, respectively. Therefore, we can conclude that the peptides CgR2150 and CgR3101 are basically non-toxic to mammals.

### 2.9. Destructive Effects of CgR2150 and CgR3101 on X. oryzae pv. oryzae and C. michiganensis

**Figure 12:**
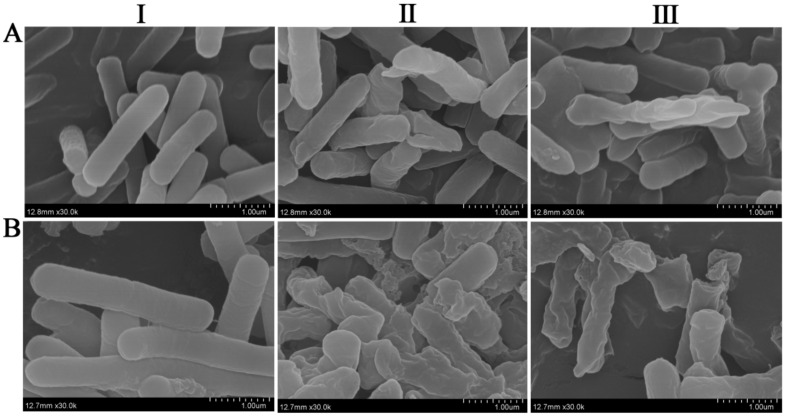
Effects of antimicrobial peptides CgR2150 and CgR3101 on cell membrane damage of *X. oryzae* pv. oryzae and *C. michiganensis* after treatment at MIC concentrations. (**A-I**) PBS, (**A-II**) CgR2150, and (**A-III**) CgR3101 acting on the cell membrane of *X. oryzae* pv. oryzae. (**B-I**) PBS, (**B-II**) CgR2150, and (**B-III**) CgR3101 acting on the cell membrane of *C. michiganensis*.

To see how these two peptides destroyed the pathogenic bacteria’s cell membrane, we examined cell samples of *X. oryzae* pv. oryzae and *C. michiganensis* treated with CgR2150 and CgR3101 under a scanning electron microscope. The results showed that the surface of the cells in the control group was smooth, complete, and normal in shape. In contrast, after treatment with peptides CgR2150 and CgR3101, the cell membrane surface of *X. oryzae* pv. oryzae was significantly shrunken and morphologically abnormal; while the cell membrane surface of *C. michiganensis* was severely damaged, with obvious gaps and ruptures (Figure 12). These results indicated that the peptides CgR2150 and CgR3101 had certain damaging effects on the cell membranes of *X. oryzae* pv. oryzae and *C. michiganensis*.

## 3. Discussion

Endophytes are a large group of microorganisms that live in plant tissues but do not cause significant damage in plants [36,37,38]. The distribution of endophytes is universal, and a large number of endophytes can be isolated from different plant parts, including plant roots, stems, leaves, flowers, fruits, tubers, seeds, and nodules [39,40,41,42,43]. Plant endophytes can produce many secondary metabolites, such as flavonoids, terpenoids and so on, which are beneficial to the prevention and control of plant pests and human diseases, so they have broad application prospects [44,45,46,47,48]. A *C. globosum* strain isolated from potato tubers in this experiment is a common endophytic fungus. It has been reported in literature that it can effectively inhibit *R*. *solani*, *F*. *oxysporum*, and *B*. *cinerea* [29,49]. In our study, it was also demonstrated that the *C. globosum* significantly inhibited the mycelial growth of *V*. *dahliae*, *F*. *oxysporum*, *R*. *solani*, *F*. *graminearum*, and *B*. *cinerea*, a species of biological control agent with application potential.

In recent years, the scope of antibiotic usage has expanded, resulting in an increasing number of drug-resistant bacteria, which poses a serious threat to human health [50]. Antimicrobial peptides are considered one of the possible therapeutics to overcome these resistant patterns because of their significant antimicrobial characteristics [51,52]. In addition, some studies have shown that antimicrobial peptides can be used in combination with existing antibiotics, which not only exerts synergistic effects, but also prevents the generation of drug resistance [53,54]. Since *B. subtilis* has the ability to secrete and express efficiently, it is easier to obtain the secreted and expressed products of exogenous genes. However, the accumulation of exogenous gene expression and secretion products to a certain extent may have toxic effects on the host cells, leading to the rupture of the host cells, and finally the phenotype of the recombinant strains will change significantly, resulting in autolysis [55,56]. Using the *B. subtilis* expression system, we have developed a novel, high-throughput technique for the isolation of novel AMPs [20,21,22,23]. In this study, we screened 10 candidate genes from 4280 clones of *C. globosum* cDNA library, and finally we selected two antimicrobial peptide genes *CgR2150* and *CgR3101* with strong antifungal and antibacterial activities for further study.

Some AMPs have been reported to show strong antibacterial activity against a variety of microorganisms and have stable physicochemical properties, so they have been used in clinical trials, including hLF-1-11, pexiganan, omiganan, and so on [57,58,59]. Since the structure, isoelectric point, and hydrophilicity of antimicrobial peptides can affect the bacteriostatic activity of AMPs [60,61], we first translated the nucleotide sequences of AMPs into amino acid sequences, and performed biological information prediction for CgR2150 and CgR3101 (Table 2 and Table 3). It was found that both peptides have amphiphilicity and a high isoelectric point (IP), and CgR2150 also has an α-helical structure, which is similar to the structure of reported peptides with broad-spectrum antibacterial activity. Furthermore, we used the ExPASy and PEP-FOLD3 online servers to predict functional peptides. For identifying antimicrobial functional domains, we used the antiBP server to narrow down sequences of the full-length gene [62]. Based on this analysis, we will perform further investigations to characterize the core sequence responsible for the antimicrobial activity.

Unlike traditional antibiotics, which have specific target sites, the mechanism of action of antimicrobial peptides involves multiple targets rather than a specific high-affinity target [63,64,65]. Antimicrobial peptides generally act on the cell membrane of bacteria, destroying its integrity, causing the cell contents to spill out of the cell and killing the bacteria [66,67]. In our study, observation by scanning electron microscopy revealed severe cell surface damage (shrinkage, rupture, and perforation) of bacteria treated with CgR2150 and CgR3101. Therefore, we speculate that CgR2150 and CgR3101 kill pathogens by disrupting their cell membranes. Although AMPs have broad-spectrum antibacterial activities, some studies have found that some AMPs have certain toxic effects on mammalian cells [68]. As a result, we performed a hemolytic assessment of CgR2150 and CgR3101 and showed that both peptides have very low hemolytic activity on porcine erythrocytes even at higher peptide concentrations (Figure 11). Based on our findings, we speculate that these two peptides obtained from *C. globosum* are relatively safe for animal cells. However, because our experiment is currently focused on the hemolysis of CgR2150 and CgR3101 peptides in porcine erythrocytes, we will further investigate the toxic effects of antimicrobial peptides on animal and plant cells to ensure that peptides are safe for organisms.

In our study, we observed that both peptides significantly reduced the disease index and morbidity of tomato plants treated with them (Table 1), indicating that both peptides are effective at controlling *C. michiganensis* and have promising biocontrol potential. As a result, we believe that these peptides can be developed into biocontrol agents for the prevention of plant disease in the future.

## 4. Materials and Methods

### 4.1. Isolation, Purification, and Screening of Endophytic Fungi

Healthy potato tubers were firstly cut into little pieces and rinsed with a 75% ethanol and 1% sodium hypochlorite solution. The tubers were then placed on PDA medium with streptomycin and cultivated for 3–7 days at 28 °C. The hyphae that had developed around the tuber’s edge were selected for separation and purification. We inoculated the endophytic and pathogenic fungi at two sites of 2 cm from the center of the PDA plate, respectively, and kept them at 28 °C for 2–5 days using the plate confrontation culture method. Then, calculating the inhibition rate, we observed the antagonism between the strains. The formula for calculating the inhibition rate (%) is as follows: Inhibition rate (%) = [(control pathogenic fungus colony radius − treated pathogenic fungal colony radius)/control pathogenic fungal colony radius–radius of pathogen disk] × 100.

### 4.2. Identification of Endophytic Fungi

The morphological identification technique was carried out by observing the fungal colony’s appearance features. To do so, we placed a small amount of lactic phenol cotton blue staining solution in the center of a clean glass slide, picked the endophytic fungus’ mycelium with a toothpick, and placed it on the glass slide, then covered it. The microscopic properties of the specimens were examined under a light microscope. To determine the isolates’ 5.8S rRNA gene sequence, we extracted the total genomic DNA using the CTAB technique with minor modifications [69]. The internal transcribes spacers (ITS1) (3′-TCCGTAGGTGAACCTGCGG-5′) and ITS4 (3′-TCCTCCGCTTATTGATATGC-5′) universal primers were used for polymerase chain reaction (PCR) amplification. For this analysis, a total amount of 25 μL of reaction mixture was employed. The PCR results of the 5.8S rRNA genes were sequenced by Wuhan Tianyi Huiyuan Biological Technology Co., Ltd. in Wuhan, Hubei, China. Nucleotide sequence homology enquiries were conducted via GenBank online search engine blastp (http://blast.ncbi.nlm.nih.gov/Blast.cgi, accessed on 15 November 2020). The CLUSTALW was used to make multiple sequence alignments and comparisons with the reference strain for each of the genes, and the Neighbor-joining method was employed to build the phylogenetic tree topologies by performing bootstrap values of 1000 data sets using MEGA7.0 tools [70]. In Supplementary Table S1, the relevant sequencing accession numbers were included for constructing phylogenetic tree analysis.

### 4.3. Activation of C. globosum

First, the PDA plate was covered in sterilized circular cellophane, and then *C. globosum* was inoculated at a spot 2 cm from the center. After two or three days of growth, another point was inoculated with *B. cinerea* 2 cm from the center of the PDA plate (the two points were on the same straight line). On the third day following inoculation with *B. cinerea*, total RNA from *C. globosum* was extracted.

### 4.4. Construction of C. globosum cDNA Library

The basic steps for constructing a cDNA library are found in Supplementary Method S1. Total RNA, mRNA, and cDNA images are shown in Supplementary Figure S2 and the primers used to create the *C. globosum* cDNA library and purify the peptides are listed in Supplementary Table S2.

### 4.5. Screening of Candidate Resistance Genes

We took 1 μL of bacterial solution from all the clones in the cDNA library, spotted them on the LB plate containing kanamycin (10 mg/L), and observed the morphology of the colonies every 12 h. The experiment was repeated 3 times to determine whether the autolysis phenomenon is stable.

### 4.6. Expression of Extracellular Peptides

Extracellular peptides were extracted using ammonium sulfate precipitation [22], and then their inhibitory activities against some Gram-positive bacteria (*C. michiganensis* and *C. fangii*), Gram-negative bacteria (*X. oryzae* pv. oryzae, *X. oryzae* pv. oryzicola, and *X. campestris* pv. Holcicola), and pathogenic fungi (*R. solani*, *F. graminearum*, and *B. cinerea*) were determined. *B. subtilis* was grown in 200 mL liquid LB medium containing kanamycin (10 mg/L) and incubated at 180 r/min, 37 °C for 72 h. The supernatant was then collected by centrifugation at 10,000× *g* for 20 min at 4 °C. The saturated ammonium sulfate solution was added dropwise with a 10 mL syringe to the supernatant (placed on ice), agitated slowly until the clear solution became hazy, and then stored at 4 °C overnight. The next day, the solution was placed at 10,000× *g*, centrifuged at 4 °C for 20 min, and the supernatant was discarded. The peptides were dissolved in PBS buffer (pH 7.0) and dialyzed for 48 h at 4 °C in the same PBS buffer.

### 4.7. Determination of the Inhibitory Effect of Antimicrobial Peptides on Pathogenic Bacteria and Fungi

To determine the effect of extracellular peptides on bacteria, the agar diffusion test (filter paper method) [71,72] was used. The detailed procedure is available in Supplementary Method S2. After the fungal hyphae had grown for 10 mm, 200 μL of extracellular peptide was pipetted into the well. Finally, it was placed in a 28 °C incubator for 2–4 days, and the growth diameter of the fungal hyphae was observed and measured.

### 4.8. Stability Determination of Antimicrobial Peptides

The AMPs were treated under different UV, temperature, and enzymes to verify their stability. The AMPs were treated with UV light at a wavelength of 265 nm for 0.5 h, 1 h, 1.5 h, and 2 h, respectively, and the effect of UV on the antibacterial activity of the AMPs was determined by agar diffusion test. In addition, the AMPs were treated at 25 °C, 50 °C, 75 °C, and 100 °C for 30 min, respectively, for thermal stability tests. Finally, AMPs were treated with 100 μg/mL lipase (30 °C), α-amylase (55 °C), pepsin, and trypsin (37 °C) for 1 h to determine the effect of different biological enzymes on them. Agarose diffusion experiments were performed on two Gram-positive (*C. michiganensis* and *C. fangii*) and Gram-negative bacteria (*X. oryzae* pv. oryzae and *X. oryzae* pv. oryzicola).

### 4.9. Purification of His-Tag Fusion Peptides

To purify the CgR2150 and CgR3101 peptides, we firstly inserted a 6× His tag and a protease (TEV) sequence between the signal peptide of the pBE-S vector and the target fragment, and then transformed it into *B. subtilis* SCK6-e cells [23]. Then, CgR2150 and CgR3101 were purified by nickel column affinity chromatography. The detailed purification procedure is available in Supplementary Method S3.

### 4.10. Tris-Tricine SDS-PAGE and Western Blotting

In order to detect the purified AMPs by western blot, we firstly used the Tricine-SDS-PAGE kit to set up the gel, and then loaded the processed samples for electrophoresis. The overall SDS-PAGE analysis procedure can be found in Supplementary Method S4. After the electrophoresis, the stacking gel and the interlayer gel were removed, and the membrane was transferred first. After the transfer, the PVDF membrane was placed in 5% nonfat milk powder to seal for 2.5–3 h. Finally, immunoblotting was performed with anti-His-tag monoclonal antibody, HRP, and ECL detection reagents. The molecular weights of AMPs were predicted by comparison with color pre-dyed low peptide molecular weight markers.

### 4.11. Determination of the Minimum Inhibitory Concentration (MIC)

The indicator bacteria were inoculated in LB liquid medium (OD600 ≈ 0.8–1.0) and then diluted with the indicator bacteria (OD600 ≈ 0.02–0.05). After that, we added 40 μL of diluted indicator bacteria solution to 96-well titer plates. The AMPs were diluted through a series of concentration gradients, and then an equal volume of peptides was added to the above 96-well plate and incubated at 28 °C for 12 h. The MIC value is the lowest concentration of peptide that significantly inhibits bacterial growth. To determine the time-kill assay, the purified antimicrobial peptide concentration was adjusted to 2× MIC concentration, and then added to a 96-well plate in equal volume with the indicator bacteria, with at least 3 wells for each concentration. After mixing the liquid evenly, it was incubated at 28 °C for 2 h, 4 h, 6 h, 8 h, 10 h, 12 h, and 24 h, and then the OD value was obtained at 600 nm.

### 4.12. Hemolytic Assay

Fresh porcine erythrocyte cells were centrifuged at 4 °C for 10 min (1500× *g*), then the cells were washed three times with pre-cooled PBS solution, and the pellet was collected and then diluted with PBS buffer. Equal volumes of diluted red blood cells and antimicrobial peptide solution (1× MIC, 2× MIC, 3× MIC, 4× MIC, and 5× MIC) were mixed and incubated at 37 °C for 1 h, centrifuged at 4000× *g* for 10 min. The supernatant was added to a 96-well plate, and the absorbance at 385 nm was measured using an ELISA plate reader. The erythrocyte suspension treated with 0.1% Triton X-100 was used as a positive control, and the suspension incubated with PBS buffer was used as a negative control. The calculation formula of the percentage of hemolytic activity is as follows: hemolysis (%) = [(A_peptide_ − A_PBS_)/(A_Triton_ − A_PBS_)] × 100.

### 4.13. Scanning Electron Microscope Analysis

*X. oryzae* pv. oryzae and *C. michiganensis* were grown at 28 °C to logarithmic growth phase. Antimicrobial peptides at 2× MIC concentration were co-incubated with bacteria for 2 h at 28 °C, and the autolysed strains (CgR2150 and CgR3101) and *B. subtilis* empty vector strain SCK6-e were shaken at 37 °C for 60 h, and then centrifuged at 1000× *g* for 5 min. After washing the cells 3 times with pre-cooled PBS (pH 7.4), they were centrifuged at 5000× *g* for 5 min and we then discarded the supernatant. The cell pellets were resuspended in 2.5% glutaraldehyde for 2–4 h to fix the cells, washed twice with PBS, and then washed with different gradients of ethanol (30%, 50%, 70%, and 90%). Finally, the samples were washed with 100% ethanol for 10 min, dried in a freeze dryer overnight, and detected by a HITACHI S-4800 SEM.

### 4.14. Pathogenicity Assay

*C. michiganensis* was cultured to the logarithmic growth phase, then the pathogenic bacteria were mixed with an equal volume of antimicrobial peptides at 2× MIC, and finally tomato plants were inoculated using the stem acupuncture method. The positive control was mixed with an equal volume of polymyxin B and pathogenic bacteria, and the negative control was mixed with an equal volume of PBS buffer and pathogenic bacteria. After inoculation, the tomato plants were cultured under greenhouse conditions, and the disease incidence was observed and recorded. The equation of disease grading criteria, incidence rate, and disease index are found in Supplementary Method S5.

## Figures and Tables

**Table 1:** Control effects of peptides CgR2150 and CgR3101 on *C. michiganensis*.

Treatment Group	Disease Index	Incidence (%)	Control Efficacy (%)
PBS Buffer	78.33 a	86.67 a	-
Polymyxin B	3.33 c	10.00 d	95.75 a
CgR2150	5.83 bc	13.33 c	92.56 b
CgR3101	7.50 b	16.67 b	90.43 c

Note. Different letters denote differences at the *p* < 0.05.

**Table 2:** Amino acid sequences of CgR2150 and CgR3101 peptides.

Peptides	Amino Acid Sequence	Accession Number
CgR2150	LAVHHLHSIRGRHHSCTIAQTQANHRHNSYQPNNSSCLAKETDLPTTRSTPATTSSTAPARKSPPASPAPTRQRLCPSPPRGPVSSRHPRRRGQARGRTRAAGVRRTKGYGGKCV	XP_001222255.1
CgR3101	LAVPNFEPPTRTTCPAPTSPGNPCGPPPPASQTRRPPFHPTSPLRR	XP_001225253.1

**Table 3:** Bioinformatics prediction of antimicrobial peptides CgR2150 and CgR3101.

Antimicrobial Peptides	Amino Acid Number	Second Structure	PI	MV
CgR2150	115	α-helix Random coil	11.96	12,469.01
CgR3101	46	Random coil	11.40	4899.59

Note, PI: theoretical pI, MV: molecular weight.

**Table 4:** MIC values (µg/mL) of CgR2150 and CgR3101 against different bacteria.

Strain	MIC (µg/mL)	
	CgR2150	CgR3101
*X. oryzae* pv. oryzae	10	12
*X. oryzae* pv. oryzicola	10	12
*C. fangii*	15	15
*X. campestris* pv. Holcicola	25	24
*C. michiganensis*	18	18

Note. Gram-positive bacteria: *C. michiganensis* and *C. fangii*. Gram-negative bacteria: *X. oryzae* pv. oryzicola, *X. oryzae* pv. oryzae, and *X. campestris* pv. Holcicola.

## Data Availability

Not applicable.

## Supplementary Materials

The following supporting information can be downloaded at: https://www.mdpi.com/article/10.3390/ijms23094611/s1.
