# Supplementary material for: Isolation of Potato Endophytes and Screening of Chaetomium globosum Antimicrobial Genes
Source: Int J Mol Sci. 2022 Apr 21;23(9):4611. doi: 10.3390/ijms23094611 (PMC9099842; doi:10.3390/ijms23094611)
Supplement: Supplementary file 1 [file ijms-23-04611-s001.zip › ijms-1662034-supplementary.pdf]

Supplementary Materials

Isolation of potato endophytes and screening of *Chaetomium globosum* antimicrobial genes

Jiaxin Zhang, Md. Samiul Islam, Jieyu Wang, Yang Zhao, and Wubei Dong*

Table S1 The list of species and GenBank accession numbers of DNA sequences for constructing phylogenetic tree

Species	Strain	Host	Location	GenBank Accession number
Chaetomium globosum	F5	Potato	China	OM929184.1
Chaetomium globosum	CBS 164.62	Unknown	Netherlands	KM655334.1
Chaetomium globosum	812-1	Pepper	Brazil	MZ648237.1
Chaetomium globosum	SVCUC14	Soil	Egypt	MG885819.1
Chaetomium globosum	TNAU Chg4	Potato	India	MK791715.1
Chaetomium jodhpurunse	Old1-1	Sunflower	China	KT192285.1
Chaetomium murorum	1-15	Asian Spicebush	China	JF502431.1
Chaetomium caprinum	NRRL 1867	Soil	USA	GU183107.1
Chaetomium sp.	72	Soyabean	China	MN871632.1
Chaetomium thermophilium	1CHP	Soil	India	EF550983.1

Chaetomium longicolleum	IBT 41567	Soil	Denmark	KX690131.1
Chaetomium trigonosporum	TTZI-8	Tuber	China	KX009483.1
Chaetomium trigonosporum	TTZI-3	Tuber	China	X009486.1
Achaetomium globosum	CBS 332.67	Soil	India	MH870682.1

Table S2 Primers for *C. globosum* library construction and target gene amplification

Primer	Sequence
Oligo dT (contains *Xba* I enzyme site)	ACAGGCTCTAGAGCTTTTTTTTTTTTTTTTTTTT T
PBE-S-F	GTTATTTCGAGTCTCTACGG
PBE-S-R	TAACCAAGCCTATGCCTACA
2150R	CGAGTTCTAGATTACACACACTTCCCCCC
3101R	CGAGTTCTAGATCATCTCCTCAGCGGAGA
F1	GGCCGGTGCACATCACCATCATCACCACG AA AACCTGTATTTTCAGTCCCA
R1	TATGGGACTGAAAATACAGGTTTTCGTGG TG ATGATGGTGATGTGCACC

Note: 2150R and 3101R are downstream sequences where the Poly A and His-tag of the original strain were cut. F1 and R1 are the sequences where the target segment (*Nde* I-His-tag-TEV-*Xba* I) was inserted.

Table S3 Amino acid sequences of antimicrobial peptides CgR2150 and CgR3101

antimicrobial peptide	Amino acid sequence	AA
CgR2150	LAVHHLHSIRGRHHSCTIAQTQANHRHNSYQPNNSSCLAKE TDLPTTRSTPATTSSTAPARKSPPASPAPTRQRLCPSPPRGPVSS RHPRRRGQARGRTRAAGVRRTKGYGGKCV	115
CgR3101	LAVPNFEPPTRTTCPAPTSPGNPCGPPPPASQTRRPPFHPTSPL RR	46

Note, AA No.: number of amino acids

Table S4 Bioinformatics prediction of antimicrobial peptides CgR2150 and CgR3101

antimicrobial peptide	Second structure	PI	MV
CgR2150	α-helix Random coil	11.96	12469.01
CgR3101	Random coil	11.40	4899.59

Note, PI: theoretical pI, MV: molecular weight.

Table S5 The bacteriostatic spectrum of the antimicrobial genes of *C. globosum*

Gene	***G***$^{+}$		***G***$^{-}$	
	C. fangii	*C. michiganensis*	*X. oryzae* pv. oryzicola	*X. oryzae* pv. oryzae
SCK6-e	0.24 ± 0.023	0.28 ± 0.057	0.31 ± 0.018	0.26 ± 0.022
CgR2150	1.21 ± 0.037 **	1.19 ± 0.033**	1.29 ± 0.034**	1.39 ± 0.036**
CgR2185	1.21 ± 0.033**	1.17 ± 0.033**	1.24 ± 0.034**	1.36 ± 0.027**
CgR3037	1.06 ± 0.033**	0.79 ± 0.033**	0.86 ± 0.024**	0.84 ± 0.036**
CgR3101	1.15 ± 0.029**	1.04 ± 0.033**	1.26 ± 0.024**	1.19 ± 0.036**
CgR2668	0.46 ± 0.037**	1.01 ± 0.033**	0.68 ± 0.027**	0.74 ± 0.034**
CgR3766	0.73 ± 0.029**	0.58 ± 0.028**	0.55 ± 0.021**	0.83 ± 0.027**
CgR987	0.56 ± 0.033**	-	0.69 ± 0.029**	0.83 ± 0.036**

CgR2616	0.57 ± 0.039**	0.52 ± 0.033**	0.57 ± 0.023**	0.54 ± 0.032**
CgR3524	0.46 ± 0.033**	0.55 ± 0.025**	0.59 ± 0.029**	0.64 ± 0.036**
CgR3815	0.26 ± 0.035	0.39 ± 0.027*	0.38 ± 0.024*	0.47 ± 0.27**

Note, "G^+" means gram-positive bacteria, "G^-" means gram-negative bacteria. "-" indicate no significant difference; * indicates significant difference (*p*≤0.05), ** indicates an extremely significant difference (*p*≤0.01). The data in the table are the mean (cm) ± standard deviation (cm) of antimicrobial diameter mean. The results are from three independent experiments. For significance analysis, t-tests were performed.

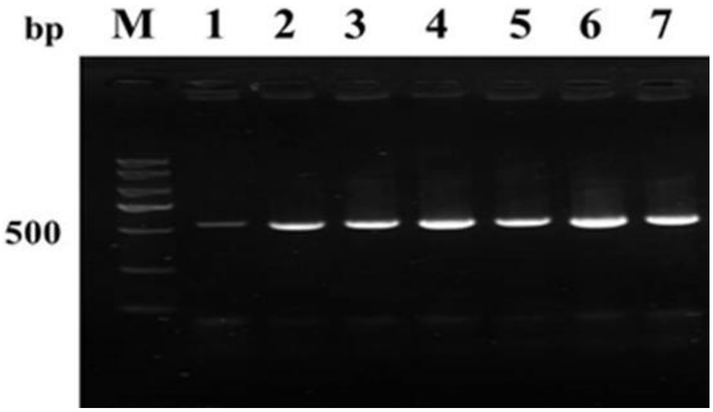

Figure S1 PCR products of *C. globosum* F5 which were amplified of the 5.8S rRNA gene sequence with the universal primer pairs (ITS1/ITS4) (500 bp). M: DL2000 bp molecular marker.

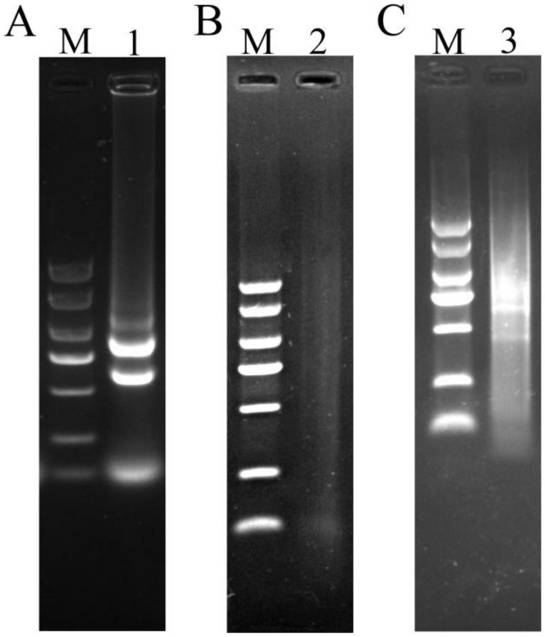

Figure S2 Quality assessment of total RNA, mRNA, and cDNA of *C. globosum*. Lane M indicates the DL2000 DNA marker. Lanes 1, 2, and 3 indicate the total RNA, mRNA, and cDNA, respectively.

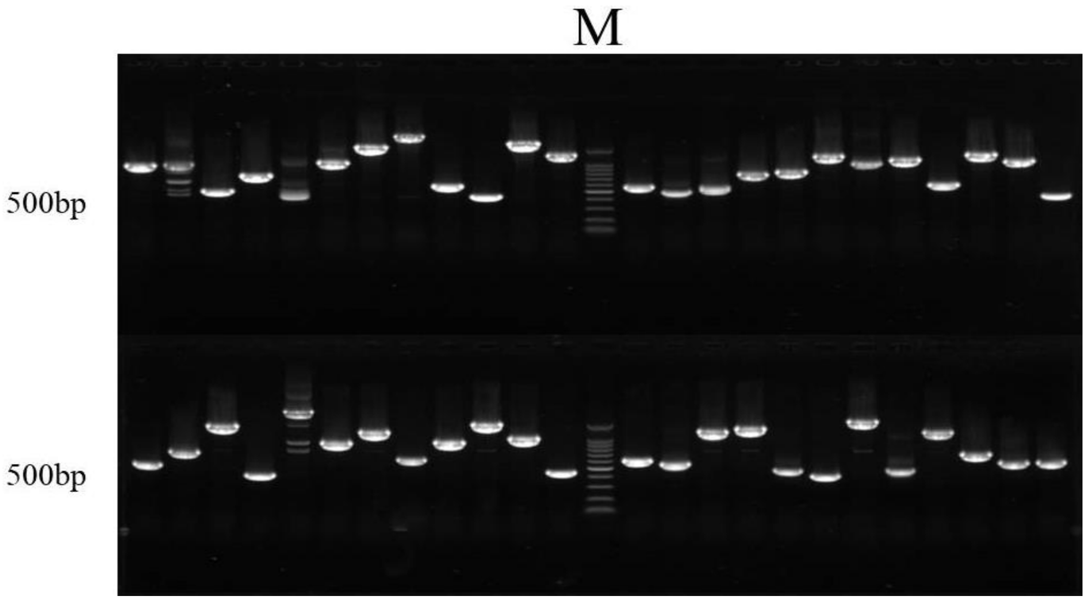

Figure S3 Insertion of the cDNA library of *B. subtilis* system. The marker was 100 bp.

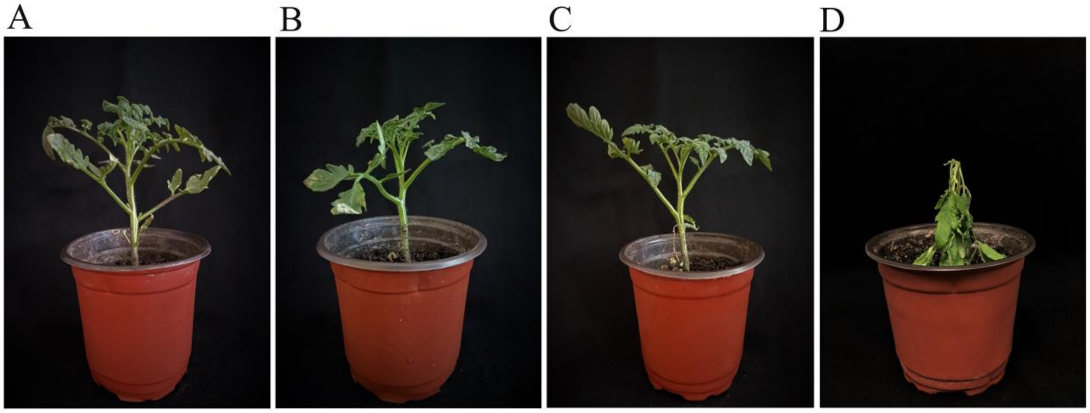

Figure S4 Biocontrol effects of antimicrobial peptides on *C. michiganensis***.** Antibacterial peptides CgR2150 and CgR3101 can effectively prevent the occurrence of *C. michiganensis*. PBS buffer treatment was used as a negative control, and polymyxin B treatment was used as a positive control. (**A**) Polymyxin B, (**B**) CgR2150, (**C**) CgR3101, and (**D**) PBS buffer show the incidence of *C. michiganensis*.

Supplementary method S1

cDNA library construction procedure:

The detailed methods are as follows: total RNA was extracted from *C. globosum* using the TRIzol method [70], mRNA was purified using PolyATtractR mRNA isolation systems (Promega, Madison, WI, USA), and cDNA was synthesized using a cDNA kit (Takara Biomedical Technology, Dalian, China). The cDNA was ligated to an adaptor containing an *Nde* I cleavage site by T_4 ligase. Both the obtained cDNA and the vector pBE-S were double digested with *Nde* I and *Xba* I, and then the digested cDNA and the vector pBE-S were ligated with T_4 ligase. The next day, the ligation product was transformed into *E. coli* TSC01 cells, and the population plasmid was extracted. Finally, the plasmid was transformed into *B. subtilis* SCK6 cells. Pick all single colonies and place them in 2 ml Eppendorf tubes in LB medium containing kanamycin (10 mg/L) and incubate at 37 °C in a shaker at 180 rpm for 4-6 h. The pBE-S-F and pBE-S-R primers were used to confirm the quality of the cDNA library and were then saved at −80 °C.

Supplementary method S2

Determination of inhibition activity

The indicator bacteria were mixed with semisolid NA medium and poured onto NA plates prepared in advance. Sterile filter papers were placed on the designated position on the agar

plate, and then 20 μL of extracellular peptides were dropped on each filter paper. Finally, all plates were incubated at 28°C for 5-7 h, and the diameter of the inhibition zone was observed and measured. The antagonism effect of extracellular peptides on fungi was determined by Oxford cup method. Two holes with a diameter of about 5 mm were formed at 25 mm from the center of the solid PDA plate using an Oxford cup, and the pathogenic fungi were placed in the center of the PDA plate respectively.

Supplementary method S3

Peptide purification procedure

B. subtilis was grown in 300 mL liquid LB medium containing kanamycin (10 mg/L) and incubated at 180 r/min, 37 °C for 72 h. The supernatant was collected by centrifugation at 10,000× g for 25 min at 4°C. The target peptides containing 6×His tags were captured by a nickel column at 4°C, and then eluted with imidazole at 600 mmol/L. The eluted target peptide was digested with TEV protease overnight at 4°C, and the next day, the solution was passed through Ni-NTA His Bind Resin, and the effluent was collected for subsequent experiments.

Supplementary method S4

SDS-PAGE electrophoresis

First, 20 μL of purified peptide was mixed with 5 μL of 5× loading buffer, and then heated at 100°C for 6-10 min. After the sample was fully denatured, it was cooled to room temperature, and then centrifuged at 12,000×g for 2 min to remove precipitated impurities. Use the Tricine-SDS-PAGE kit to configure the gel, wait for the gel to solidify, pipette 25 μL of the treated sample into the gel well, and then perform electrophoresis. After electrophoresis, use Coomassie brilliant blue for staining, and finally decolorize with decolorizing solution until the protein gel band and background are both clear.

Supplementary method S5

Pathogenecity assay equation

C. michiganensis disease grading criteria are as follows, grade 0: no wilting or stem necrosis of leaves (or cotyledons); grade 1: mild yellowing or wilting of leaves (or cotyledons); grade 2: moderate wilting or wilting of leaves (or cotyledons) and mild necrosis of stem; Grade 3: severe wilting of leaves (or cotyledons) or severe necrosis of stem; Grade 4: plant death. The formulas for incidence rate, disease index and control efficacy are as follows: Incidence rate

(%) = (number of diseased plants/number of inoculated plants) × 100. Disease index = [Σ (value of disease grade × number of plants of this disease grade)]/(highest value of disease grade × total number of investigated plants) × 100. Control efficacy (%) = (the disease index of the control group - the disease index of the treatment group) / the disease index of the control group × 100.
